# Efficacy and safety of low dose aspirin and magnesium sulfate in the treatment of pregnancy induced hypertension: A protocol for systematic review and meta-analysis

**DOI:** 10.1097/MD.0000000000022801

**Published:** 2020-11-13

**Authors:** Guolin He, Yihong Chen, Meng Chen, Guoqian He, Xinghui Liu

**Affiliations:** a Key Laboratory of Birth Defects and Related Diseases of Women and Children, Ministry of Education, West China Second University Hospital, Sichuan University; b Department of Obstetrics and Gynecology, West China Second University Hospital, Sichuan University, Chengdu, Sichuan, China.

**Keywords:** low dose aspirin, magnesium sulfate, meta-analysis, pregnancy induced hypertension, protocol, systematic review

## Abstract

**Background:**

Magnesium sulfate combined with low-dose aspirin can significantly reduce adverse reactions and effectively lower blood pressure in patients with pregnancy induced hypertension, but the overall efficacy and safety of the combination of drugs are not clear. The purpose of this study was to evaluate the efficacy and safety of magnesium sulfate combined with low-dose aspirin in the treatment of pregnancy induced hypertension.

**Methods:**

Randomized controlled trials focusing on the administration of magnesium sulfate combined with low-dose aspirin for pregnancy induced hypertension were searched from PubMed, EMbase, Cochrane Library, Web of Science, China National Knowledge Infrastructure (CNKI), WanFang, and the Chongqing VIP Chinese Science and Technology Periodical Database. Two researchers independently screened titles, abstracts, and full texts, and extracted relevant data. The RevMan 5.3 software and Stata 14 software were used for statistical analysis.

**Results:**

The effect and safety of magnesium sulfate combined with low-dose aspirin in the treatment of pregnancy induced hypertension were assessed by summarizing the related randomized controlled trials.

**Conclusion:**

This article provides theoretical support for the clinical application of magnesium sulfate combined with low-dose aspirin in the treatment of pregnancy induced hypertension.

**OSF Registration number:**

DOI 10.17605/OSF.IO/SZFGB

## 1 Introduction

Pregnancy induced hypertension is a disease that occurs in women during pregnancy, featured by systolic blood pressure greater than 140 mm Hg or / and diastolic blood pressure greater than 90 mm Hg.^[1–3]^ Its clinical manifestations include elevated blood pressure, proteinuria, cardiac and renal insufficiency in severe cases. What is worse, it even threatens the life safety of fetuses and parturient women. The incidence of pregnancy induced hypertension in China is about 9.4% and 10.1%.^[4]^

Magnesium sulfate is the first choice for the treatment of pregnancy induced hypertension in obstetrics due to its spasmolytic, sedative, and antihypertensive effects.^[5–7]^ However, an overdose of magnesium sulfate can cause side effects such as respiratory paralysis, and it is not applicable to patients with cardiac and renal insufficiency.^[8]^ Recent studies have shown that magnesium sulfate combined with low-dose aspirin can markedly reduce adverse reactions and effectively bring the high blood pressure down^[9]^ in patients with pregnancy induced hypertension. In this study, a meta-analysis was made to systematically evaluate the efficacy and safety of magnesium sulfate combined with low-dose aspirin in the treatment of pregnancy induced hypertension, so as to provide evidence-based medicine for the treatment and medication of pregnancy induced hypertension.

## 2 Methods

### 2.1 Protocol registration

The protocol of this study has been drafted under the guidance of the Preferred Reporting Items for Systematic Reviews and Meta-analyses Protocols (PRISMA-P).^[10]^ Moreover, it has been registered on OSF (Registration number: DOI 10.17605/OSF.IO/SZFGB).

### 2.2 Ethics

Ethical approval is not required because there is no patient recruitment or personal information collection, and the data included in our study were extracted from published literature.

### 2.3 Inclusion criteria for research programs

#### 2.3.1 Type of studies

Randomized controlled trials in English or Chinese on the application of magnesium sulfate combined with low-dose aspirin in the treatment of pregnancy induced hypertension were included.

#### 2.3.2 Study object

The study cohort was composed of patients diagnosed with pregnancy induced hypertension.

#### 2.3.3 Intervention type

The experimental group was treated with magnesium sulfate combined with low-dose aspirin, and the control group was treated with magnesium sulfate only.

#### 2.3.4 Outcome measurements

Primary outcome: clinical effective rate.

Secondary outcomes:

(1) hematocrit;
(2) blood viscosity;
(3) 24 hours urinary protein;
(4) adverse reactions.

### 2.4 Exclusion criteria

(1) Articles published as abstracts or conference papers and whose data could not be extracted by contacting the author were excluded;
(2) Repeatedly published articles were eliminated, but research projects with the most complete information were input;
(3) Articles with either incomplete or erroneous original data were removed;
(4) Articles with obvious randomization mistakes were also excluded.

### 2.5 Search strategy

Randomized controlled trials on the application of magnesium sulfate combined with low-dose aspirin in the treatment of pregnancy induced hypertension were retrieved from PubMed, EMbase, Cochrane Library, Web of Science, CNKI, WanFang, and the Chongqing VIP Chinese Science and Technology Periodical Database. The retrieval words used were: magnesium sulfate, aspirin, pregnancy induced hypertension, gestational hypertension, pregnancy induced hypertension, etc. PubMed retrieval strategies are displayed in Table 1. We also adapted similar search strategies for other electronic databases.

**Table 1:** Search strategy in PubMed database.

Number	Search terms
#1	Hypertension, Pregnancy-Induced[Mesh]
#2	Gestational Hypertension[Title/Abstract]
#3	Transient Hypertension, Pregnancy[Title/Abstract]
#4	Pregnancy Induced Hypertension[Title/Abstract]
#5	Hypertension, Gestational[Title/Abstract]
#6	Hypertension, Pregnancy Induced
#7	Hypertension, Pregnancy Transient[Mesh]
#8	Hypertensions, Pregnancy Induced[Title/Abstract]
#9	Induced Hypertension, Pregnancy[Title/Abstract]
#10	Induced Hypertensions, Pregnancy[Title/Abstract]
#11	Pregnancy Transient Hypertension[Title/Abstract]
#12	Pregnancy-Induced Hypertension[Title/Abstract]
#13	#1 OR #2 OR #3 OR #4 OR #5 OR #6 OR #7 OR # OR 8# OR 9# OR 10# OR 11# OR 12#
#14	Aspirin[Mesh]
#15	Acetylsalicylic Acid[Title/Abstract]
#16	2-(Acetyloxy)benzoic Acid[Title/Abstract]
#17	Acetysal[Title/Abstract]
#18	Acylpyrin[Title/Abstract]
#19	Aloxiprimum[Title/Abstract]
#20	Colfarit[Title/Abstract]
#21	Dispril[Title/Abstract]
#22	Easprin[Title/Abstract]
#23	Ecotrin[Title/Abstract]
#24	Endosprin[Title/Abstract]
#25	Magnecyl[Title/Abstract]
#26	Micristin[Title/Abstract]
#27	Polopirin[Title/Abstract]
#28	Polopiryna[Title/Abstract]
#29	Solprin[Title/Abstract]
#30	Solupsan[Title/Abstract]
#31	Zorprin[Title/Abstract]
#32	Acid, Acetylsalicylic[Title/Abstract]
#33	#14 OR #15 OR #16 OR #17 OR #18 OR #19 OR #20 OR #21 OR #22 OR #23 OR #24 OR #25 OR #26 OR #27 OR #28# OR #29 OR #30 OR #31 OR #32
#34	Magnesium Sulfate[Mesh]
#35	Magnesium Sulfate, Heptahydrate[Title/Abstract]
#36	Heptahydrate Magnesium Sulfate[Title/Abstract]
#37	Sulfate, Magnesium[Title/Abstract]
#38	#34 OR #35 OR #36 OR #37
#39	#13 AND #33 AND #38

### 2.6 Data extraction principle

**Figure 1:**
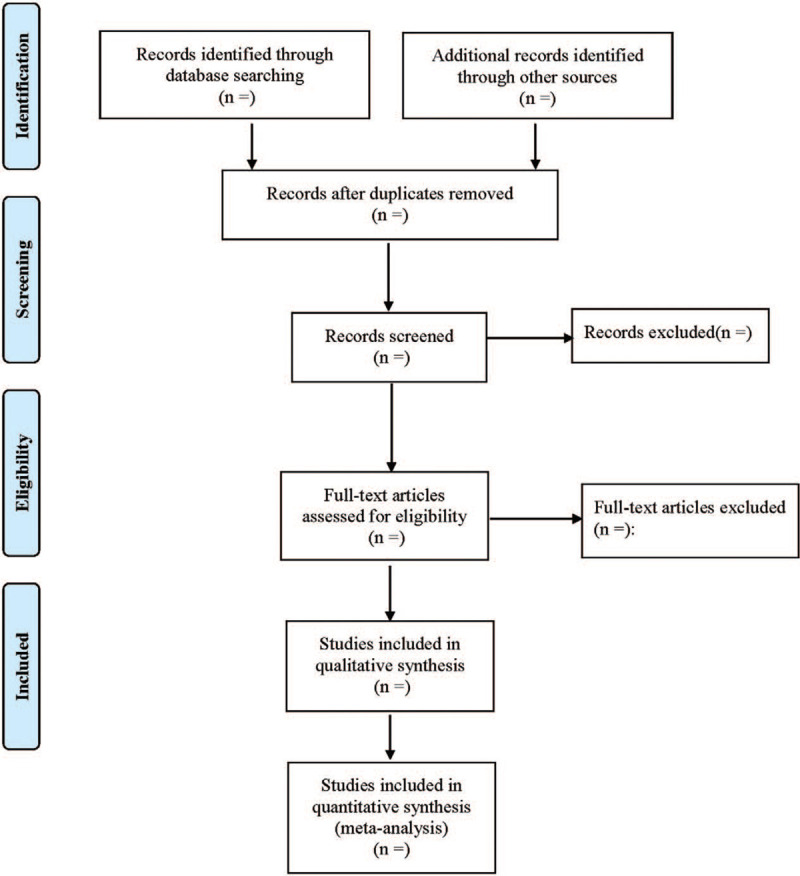
Flow diagram of study selection process.

Two investigators independently screened the articles. First, duplicate pieces of literature were eliminated. Second, literature that did not meet the inclusion criteria were excluded by reviewing the titles and abstracts. Third, the full text of the literature that might meet the inclusion criteria was rescreened to determine whether it was finally included or not, and then a cross-check was made. Any disagreement was solved via dialogues or discussion with a third investigator when necessary. The 2 investigators extracted the data and cross-checked them separately. Excel 2019 literature information database was established to extract data including the author(s), year of publication, sample size, drug dose, sex, age, intervention measures, course of treatment, outcome, etc. In case of disagreement, the issue was discussed and resolved with a third investigator. The literature selection process is illustrated in Figure 1.

### 2.7 Literature quality evaluation

The 2 researchers independently assessed the risk of bias in the included literature by referring to the Cochrane system reviewer's manual. The risk levels of literature quality bias were identified as high, unclear and low. If there was any disagreement, the decision was made after consultation with a third researcher.

### 2.8 Statistic analysis

#### 2.8.1 Data analysis and processing

The RevMan 5.3 software (The Cochrane Collaboration, Software Update, Oxford, UK) and Stata 14 software (STATA Corporation, College Station, TX) were used for statistical analysis. Dichotomous variables were analyzed using the relative risk, and continuous variables measured with different tools or units were studied with the Standardized mean difference. These 2 types of variables were all represented by the effect values and 95% confidence intervals. Inter-study heterogeneity was determined by the *Q* test. *P* values ≥.1 suggested no inter-study heterogeneity, while *P* values <.1 indicated there was heterogeneity among studies. At the same time, the *I*^*2*^ value was used to quantitatively evaluate the inter-study heterogeneity. If *I*^*2*^ ≤ 50%, the heterogeneity was considered to be adequate, and the fixed-effect model was adopted. *I*^*2*^ > 50% implied significant heterogeneity, and the source of heterogeneity would be explored through either subgroup analysis or sensitivity analysis. If there was no obvious clinical or methodological heterogeneity detected, statistical heterogeneity was assumed, and the random-effect model would be used for analysis. Descriptive analysis was carried out if there was significant clinical heterogeneity between the two groups and subgroup analysis was not available.

#### 2.8.2 Missing data processing

If data was reported missing or incomplete, we would ask the corresponding author for the missing pieces of information. Otherwise, the concerned study would be removed.

#### 2.8.3 Subgroup analysis

In order to eliminate the clinical heterogeneity between studies, a subgroup analysis was conducted according to the drug dose.

#### 2.8.4 Sensitivity analysis

To test the stability of the meta-analysis results, a one-by-one elimination method was adopted for sensitivity analysis.

#### 2.8.5 Reporting bias

If the included study was ≥10, the funnel plot was drawn to qualitatively detect publication bias,^[11,12]^ and Egger and Begg tests were performed to quantitatively assess the potential publication bias subsequently.

## 3 Discussion

Pregnancy induced hypertension is one of the most prevalent complications during pregnancy,^[13,14]^ usually occurring at 20 weeks of pregnancy or about 2 weeks after delivery. In the United States, 15% of maternal deaths are caused by high blood pressure and its complications.^[15]^ The etiology of pregnancy induced hypertension remains to be clarified. It is claimed to be the result of factors as diverse as the mother, fetus, placenta, etc.

Magnesium sulfate has become the first-line drug for the treatment of pregnancy induced hypertension because of its good antispasmodic, sedative, and antihypertensive effects.^[16]^ Magnesium ions in magnesium sulfate can inhibit the release of acetylcholine, thus blocking the conduction between nerves and muscles and relaxing skeletal muscles.^[17]^ Magnesium ions can also dilate blood vessels, lower blood pressure and eliminate edema by preventing the sympathetic nerve from transmitting signals.^[18]^ Magnesium sulfate is capable of effectively inhibiting the synthesis of endothelin, relieving vasospasm and improving the affinity of hemoglobin between the mother and infant by stimulating prostaglandins in blood vessels.^[19]^ However, excessive use of magnesium sulfate will lead to side effects such as reduction of urine volume, disappearance of knee tendon reflection, arthralgia in exhalation, cardiorenal insufficiency, and so on.^[20]^ Aspirin is distributed throughout the body in the form of salicylic acid after hydrolysis.^[21]^ By regulating the ratio of prostaglandin to thromboxane, low-dose aspirin reduces the sensitivity of blood vessels to vasoconstrictor substances and inhibits the production of cyclooxygenase. As a result, the synthesis of thromboxane was reduced, blood vessels were relaxed and platelet aggregation was suppressed.^[22]^ The use of low-dose aspirin can create favorable conditions for magnesium sulfate to quickly relieve symptoms. Therefore, the combination of magnesium sulfate and low-dose aspirin can improve the therapeutic effect and reduce the occurrence of adverse reactions. Based on the existing evidence, this study systematically evaluates the efficacy and safety of magnesium sulfate combined with low-dose aspirin in the treatment of pregnancy induced hypertension. The results will provide better choices and more convincing evidence for the clinical treatment of the disease.

There are some limitations in this study. The literature included in this study is either in Chinese or in English, which may have an impact on the results due to the differences in the lifestyle and eating habits between China and foreign countries. Moreover, the quality of the literature is low, the drug use schemes are different, and the risk of bias is high. Therefore, the conclusions of this study should be verified by more high-quality clinical studies with a larger sample size.

## Author contributions

**Conceptualization:** Xinghui Liu.

**Funding support:** Xinghui Liu.

**Literature retrieval:** Yihong Chen.

**Methodology:** Yihong Chen.

**Software:** Meng Chen.

**Supervision:** Guoqian He.

**Writing – original draft:** Xinghui Liu, Guolin He.

**Writing – review & editing:** Xinghui Liu, Guolin He.
